# Autophagy Protects Against Developing Increased Lung Permeability and Hypoxemia by Down Regulating Inflammasome Activity and IL-1β in LPS Plus Mechanical Ventilation-Induced Acute Lung Injury

**DOI:** 10.3389/fimmu.2020.00207

**Published:** 2020-02-14

**Authors:** Nobuyuki Nosaka, Daisy Martinon, Debbie Moreira, Timothy R. Crother, Moshe Arditi, Kenichi Shimada

**Affiliations:** ^1^Division of Infectious Diseases and Immunology, Department of Pediatrics, Cedars-Sinai Medical Center, Los Angeles, CA, United States; ^2^Department of Biomedical Sciences, Cedars-Sinai Medical Center, Los Angeles, CA, United States

**Keywords:** acute lung injury, autophagy, inflammasome, IL-1β, lipopolysaccharide, macrophage, mechanical ventilation

## Abstract

Targeting inflammasome activation to modulate interleukin (IL)-1β is a promising treatment strategy against acute respiratory distress syndrome and ventilator-induced lung injury (VILI). Autophagy is a key regulator of inflammasome activation in macrophages. Here, we investigated the role of autophagy in the development of acute lung injury (ALI) induced by lipopolysaccharide (LPS) and mechanical ventilation (MV). Two hours before starting MV, 0.2 mg/kg LPS was administered to mice intratracheally. Mice were then placed on high-volume MV (30 ml/kg with 3 cmH_2_O positive end-expiratory pressure for 2.5 h without additional oxygen application). Mice with myeloid-specific deletion of the autophagic protein ATG16L1 (*Atg16l1*^fl/fl^ *LysM*^Cre^) suffered severe hypoxemia (adjusted *p* < 0.05) and increased lung permeability (*p* < 0.05, albumin level in bronchoalveolar lavage fluid) with significantly higher IL-1β release into alveolar space (*p* < 0.05). Induction of autophagy by fasting-induced starvation led to improved arterial oxygenation (adjusted *p* < 0.0001) and lung permeability (*p* < 0.05), as well as significantly suppressed IL-1β production (*p* < 0.01). Intratracheal treatment with anti-mouse IL-1β monoclonal antibody (mAb; 2.5 mg/kg) significantly improved arterial oxygenation (adjusted *p* < 0.01) as well as lung permeability (*p* < 0.05). On the other hand, deletion of IL-1α gene or use of anti-mouse IL-1α mAb (2.5 mg/kg) provided no significant protection, suggesting that the LPS and MV-induced ALI is primarily dependent on IL-1β, but independent of IL-1α. These observations suggest that autophagy has a protective role in controlling inflammasome activation and production of IL-1β, which plays a critical role in developing hypoxemia and increased lung permeability in LPS plus MV-induced acute lung injury.

## Introduction

Acute respiratory distress syndrome (ARDS) is the acute onset of non-cardiogenic pulmonary edema caused by increased pulmonary vascular permeability (1). ARDS is a major cause of respiratory failure in critically ill patients with basic inflammatory diseases, such as pneumonia, sepsis, and severe trauma (2). Mechanical ventilation (MV) is the most critical intervention in ARDS treatment, which decreases respiratory load in ARDS patients by maintaining ability of gas exchange (3). Although MV has an obvious advantage in supporting these patients, MV has also been associated with significant complications aggravating lung injury which results in increased mortality (4). This injury has been termed ventilator-induced or -associated lung injury (VILI/VALI). Reducing VILI/VALI is believed to be the key to decrease mortality in ARDS, thus elucidating the mechanisms of VILI/VALI remains a high priority research (4).

Targeting interleukin (IL)-1β is a promising treatment strategy against ARDS since IL-1β is one of the most biologically active proinflammatory cytokine in the lungs of ARDS patients (5). However, the biological mechanisms that activate the IL-1β pathway in acute lung injury (ALI) are still elusive. Alveolar macrophages (AMs) are responsible for IL-1β production in ALI (6–8). Production of IL-1β is strictly regulated and involves a two-step activation pathway (9). The ligation of pattern-recognition receptors (e.g., Toll-like receptors [TLRs]) by conserved microbial structures (e.g., lipopolysaccharide [LPS]) is the first signal which leads to production of inactive precursor of IL-1β (pro-IL-1β) (9). This pro-IL-1β then needs to be cleaved by active caspase-1 to be released in mature, biologically active form of IL-1β (9). The nucleotide binding domain and leucin-rich repeat pyrin domain containing 3 (NLRP3) inflammasome plays a crucial role for the activation of caspase-1 to produce IL-1β (9). Various groups have demonstrated that NLRP3 inflammasome is strongly associated with the development of VILI/VALI models (10–13).

Autophagy is a highly conserved, fundamental intracellular system associated with cellular homeostasis, recycling, and elimination of defective organelles, and intracellular pathogens (14, 15). The role of autophagy in the mechanism of ALI has been controversial with studies that have suggested both protective and detrimental effects of autophagy (14, 16–18). On the other hand, numerous studies have demonstrated in various settings that autophagy plays an inhibitory role in secretion of IL-1β through downregulation of the NLRP3 activation (15). These conflicting data suggest that the role of autophagy in ALI may be context dependent and should be further investigated in relevant experimental models.

During the common clinical scenario of sepsis or pneumonia plus MV, the “two-hit” lung injury model in which two independent insults (typically, bacterial lipopolysaccharide [LPS] and MV) acts synergistically to amplify the lung injury that leads to ALI/VILI (19–26). Indeed, sepsis is the main independent risk factor for ARDS in patients on MV (27). We have recently demonstrated that the LPS-plus-MV induced-mouse model of ARDS triggers extracellular ATP and subsequent NLRP3 activation followed by IL-1β release in the lungs that led to lung inflammation, severe hypoxemia as well as increased lung permeability (12). We also reported that this LPS/MV-induced hypoxemia model was dependent on NLRP3 inflammasome, and was attenuated by the IL-1R antagonist (Anakinra) which blocks both IL-1α and IL-1β (12). Since we have shown the key role of IL-1 in the induction of hypoxemia in this LPS plus MV “two-hit” lung injury model (12), and since several other prior studies have demonstrated that autophagy inhibits NLRP3 inflammasome activation and IL-1β release by LPS/ATP-treated macrophages (28–31), we then hypothesized that autophagy would play a beneficial role in the LPS plus MV ALI model.

In this study we found that mice that lack of autophagy gene *Atg16l1* specifically in macrophages, developed significantly more severe ALI; including severe hypoxemia, increased lung permeability, and greater IL-1β production in the alveolar space. On the other hand, inducing autophagy by fasting mice prior to LPS plus MV, protected the mice from ALI and hypoxemia. Finally, we observed that, while IL-1β played a critical role in developing hypoxemia in LPS + MV induced ALI, IL-1α was dispensable.

## Materials and Methods

### Mice

Wild-type (WT) C57BL/6J male mice were purchased from The Jackson Laboratory (Bar Harbor, ME, USA). *Atg16l1*^fl/fl^ mice were kindly provided by Dr. D. Q. Shih (Cedars-Sinai Medical Center, CA, USA) and bred with *LysM*^Cre^ mice (The Jackson Laboratory) (32). Littermate *Atg16l1*^fl/fl^ controls male mice were used in all *Atg16l1*^fl/fl^ *LysM*^Cre^ male mice experiments. Deletion of *Atg16l1* in addition to functional autophagy deficiency in macrophages was previously described and validated by the lack of Atg16l1 mRNA and protein, and by the lack of conversion of LC3-I to LC3-II, respectively (32, 33). *Il1*α^−/−^ mice were kindly provided by Dr. Y. Iwakura (The University of Tokyo, Tokyo, Japan) and its production was well described (34). Additional genotyping of these animals by our hands is shown in Figure S1. All mice used *in vivo* experiments were male and 8-13 weeks of age (*n* = 64 for WT mice; *n* = 12 for *Atg16l1*^fl/fl^ *LysM*^Cre^ mice; *n* = 12 for *Atg16l1*^fl/fl^ controls; *n* = 8 for *Il1*α^−/−^ mice). All animals were housed under specific pathogen-free conditions at the Comparative Medicine Facility of Cedars-Sinai Medical Center.

### Ethics Statement

All experiments were performed according to Cedars-Sinai Medical Center Institutional Animal Care and Use Committee (IACUC) guidelines.

### LPS/MV-Induced ALI Model

In this study, we utilized the LPS plus MV induced “two-hit” acute lung injury models, as we previously described (12) with minor modifications. Briefly, 2 h before starting MV, 0.2 mg/kg of LPS (O111:B4, ultrapure; InvivoGen, San Diego, CA, USA) was administered intratracheally. After anesthetized with intraperitoneal injection of 50 mg/kg of ketamine (Vedco Inc., Saint Joseph, MO, USA) and 0.5 mg/kg of dexmedetomidine (Pfizer, Irvine, CA, USA), mice were orotracheally intubated (35) and ventilated for 2.5 h using VentElite (Harvard Apparatus, Holliston, MA, USA) with a tidal volume of 30 ml/kg and a respiratory rate of 35 per minute, with 3 cmH_2_O PEEP. Inspiratory to Expiratory ratio was set as 3:7. No additional oxygen was applied. Mice were kept warm on a heating pad (Hallowell EMC, Pittsfield, MA) to maintain body temperature around 37°C.

### Anti-IL-1 Treatment

In some experiments of this study, mice were treated with different intervention in addition to the protocol described above to evaluate these effects in two-hit lung injury model. In some studies WT mice were treated with anti-IL-1α monoclonal antibody (*InVivo*MAb anti-mouse IL-1α mAb; clone ALF-161; 2.5 mg/kg; Bio X Cell, West Lebanon, NH, USA) or control IgG (*InVivo*MAb Armenian hamster IgG, Bio X Cell) intratracheally with LPS 2 h before starting MV. In other studies WT mice were treated with anti-IL-1β monoclonal antibody (*InVivo*MAb anti-mouse IL-1β mAb; clone B122; 2.5 mg/kg; Bio X Cell) or control IgG (*InVivo*MAb Armenian hamster IgG, Bio X Cell) intratracheally with LPS 2 h before starting MV. Antibody dose was chosen based on a previously published study (36, 37).

### Fasting and Trehalose Treatment

WT C57BL/6J male mice were fasted for 20 h prior to MV with free-access water (38, 39). Some mice were administered trehalose (1 g/kg, MP Biochemicals, Solon, OH, USA) intraperitoneally at the time point of 48, 24, and 2 h before starting MV. Trehalose dose was chosen based on a previously published studies (40, 41).

### Bone Marrow Derived Macrophages

Femurs and tibiae of 8–10 weeks old *Atg16l1*^fl/fl^ and *Atg16l1*^fl/fl^ *LysM*^Cre^ male mice were flushed with RPMI 1640 medium. Bone marrow cells were cultured in RPMI 1640 medium containing 10% FBS and 15% L929 cell conditioned medium with 2 media changes. BMDM were harvested on day 7 and were stimulated with LPS (1 μg/ml) for 4 h, then exposed to ATP (5 mM; Sigma-Aldrich) or Nigericin (10 μM; Enzo Life Sciences) for 45 min. The concentration of IL-1β, TNF-α, and IL-6 in the culture supernatant were determined by ELISA (eBiosciences, San Diego CA, USA and BD, Becton Dickinson, Franklin Lakes, NJ, USA).

### Arterial Blood Gas Analysis

Arterial partial pressure of oxygen was measured using the i-STAT Analyzer (Abbot Laboratories, Lake Bluff, IL, USA) at two time points during MV (30 and 150 min after MV installation). Blood was collected from anesthetized mice via the tail artery by nicking the tail with a blade. Approximately, 100 μL of whole arterial blood was collected using a heparinized capillary tube. Over-bleeding is prevented by applying a bandage.

### Bronchoalveolar Lavage (BAL)

BAL fluid (BALF) was obtained after 2.5 h MV with 0.5 mL of PBS with 2 mM EDTA by inserting a standard disposable intravenous catheter (BD Insyte Autoguard, 20GA 1.00 in., Becton Dickinson Infusion Therapy Systems Inc.) into the trachea. Cells were isolated from supernatant and analyzed for cell counts (Cellometer Auto2000, Nexcelcom Bioscience, Lawrence, MA) and differentiation by flow cytometry (SA3800 Spectral Analyzer, Sony, Tokyo, Japan). The levels of albumin and cytokines in BALF were measured using commercially available ELISA kits (albumin: Bethyl Laboratories, Montgomery, TX, USA; IL-1α: ELISA MAX, BioLegend, San Diego, CA, USA; IL-1β and TNF-α: eBioscience; IL-6: BD OptEIA, Becton Dickinson; IL-18: Sino Biological, Beijing, China) as previously described (12).

### Western Blotting

The lungs were harvested from mice and homogenized in ice-cold lysis buffer consisting of 10 mM KCl, 10 mM HEPES, 1 mM EDTA, and 1% Triton-X100. Lung homogenates were then centrifuged (10,000 g for 10 min at 4°C) to remove unbroken cells and debris. Protein quantification was performed on supernatants using bicinchoninic copper assay (Invitrogen, Carlsbad, CA). Equal masses of protein were loaded in 10–20% SDS page gels (Invitrogen) and transferred to PVDF membrane (ThermoFisher Scientific, Waltham, MA). Anti-LC3A/B (Cell Signaling Technology, Danvers, MA) and anti-β-Actin (Sigma) antibodies were diluted at a concentration of 1:1,000 and 1:10,000, respectively, in blocking buffer consisting of 5% nonfat dry milk (Bio Rad, Hercules, CA) dissolved in TBS-T. HRP-conjugated goat anti-mouse (Jackson ImmunoResearch, West Grove, PA) and anti-rabbit (Jackson ImmunoResearch) secondary antibodies were diluted at a concentration of 1:10,000 in blocking buffer. Chemiluminescent substrate (ThermoFisher Scientific) was applied to membranes, and bands were imaged via Bio-Rad Chemidoc (Bio-Rad). Densitometry of bands was performed using ImageJ 1.52q software (National Institute of Health, USA).

### Statistical Analysis

All data were analyzed with Prism 7 (GraphPad Software Inc., La Jolla, CA, USA). Comparisons were performed with the Mann-Whitney *U*-test, one-way ANOVA with Holm-Sidak's multiple comparison test, or two-way ANOVA with Sidak's multiple comparison test where appropriate. A *p* < 0.05 was considered statistically significant.

## Results

### IL-1β Is Required for LPS/MV-Induced ALI

**Figure 1:**
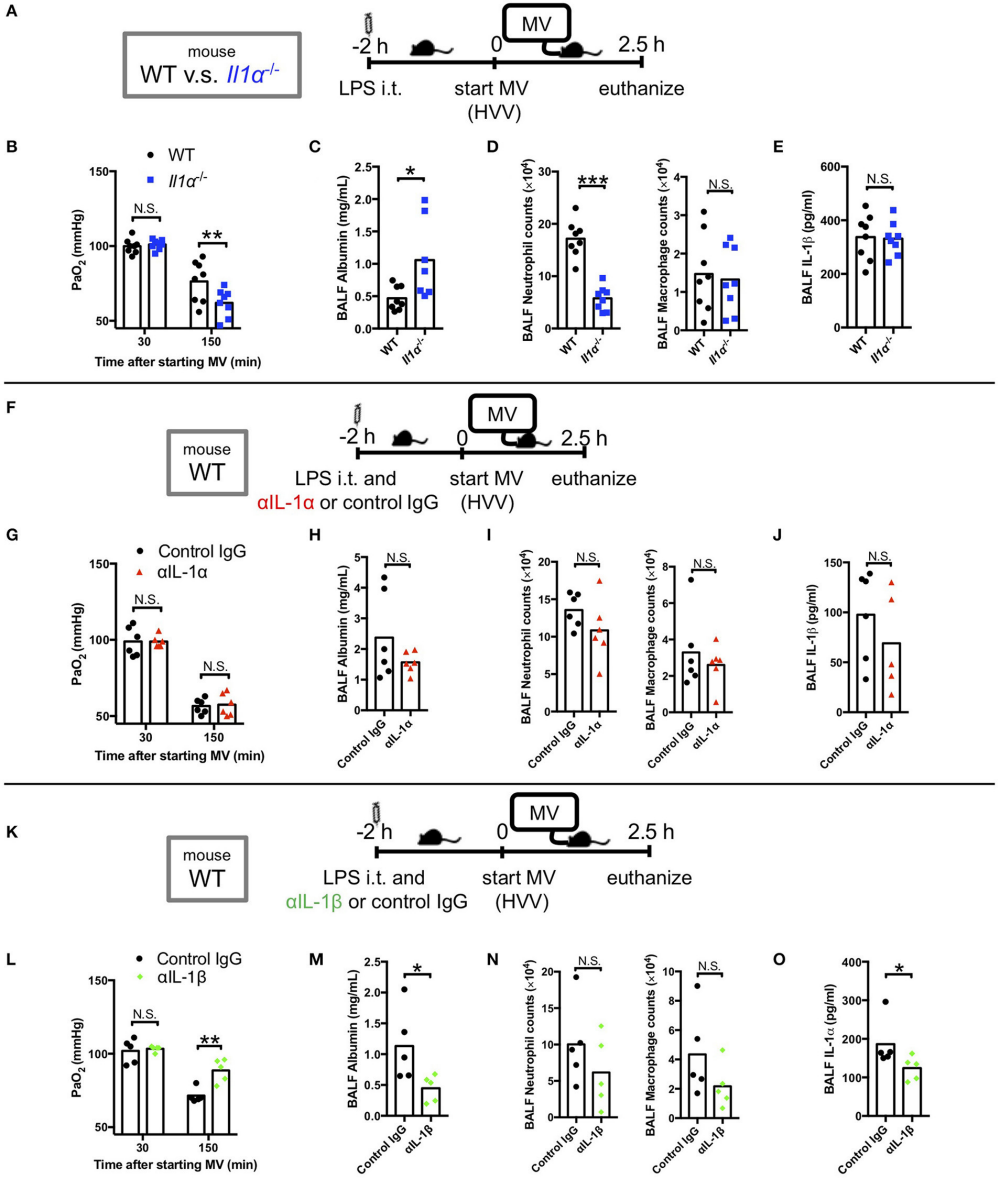
IL-1β plays a greater role in mediating lung permeability increase and hypoxemia than IL-1α. **(A–E)** The effect of conventional IL-1α deficiency in two-hit lung injury model. **(F–J)** The effect of intratracheal (i.t.) anti-IL-1α monoclonal antibody (αIL-1α) treatment in two-hit lung injury. **(K–O)** The effect of i.t. anti-IL-1β monoclonal antibody (αIL-1β) treatment in two-hit lung injury. **(A,F,K)** Study protocol. Mice were anesthetized and placed on high volume mechanical ventilation (HVV) with tidal volumes of 30 ml/kg with 3 cmH_2_O positive end-expiratory pressure (PEEP) for 2.5 h. Antibodies (2.5 μg/g) were administered i.t. at the same time with i.t. LPS. **(B,G,L)** Arterial partial pressure of oxygen (PaO_2_). PaO_2_ was measured at 30 and 150 min after starting MV. **(B,L)** **indicates significant difference (adjusted *P* < 0.01), determined by two-way ANOVA followed by Sidak's multiple comparisons test. **(G)** No significance was detected in PaO_2_ control lgG and αlL-1α mice by two-way ANOVA followed by Sidak's multiple comparisons test. **(C,H,M)** Lung permeability determined by albumin in BALF. **(D,l,N)** Absolute counts of neutrophils and macrophages in BALF. **(E,J)** IL-1β or **(O)** IL-1α levels determined in BALF. **(C,D,M)** *,***indicates *P* < 0.05, and *P* < 0.001, respectively, determined by Mann-Whitney *U*-test. N.S., not significant.

We have previously reported that LPS/MV-induced hypoxemia is dependent on NLRP3 inflammasome, and is attenuated by anakinra, the antagonist of IL-1 receptor which binds both IL-1α and IL-1β (12). Accordingly, we first sought to gain insight on the role of IL-1α in this LPS/MV-induced experimental ALI model. Intriguingly, although neutrophil infiltration into alveolar space was significantly attenuated in *Il1*α^−/−^ mice, albumin leakage in BALF was significantly higher and arterial oxygenation was worse in *Il1*α^−/−^ mice (Figures 1A–E). However, intratracheal anti-IL-1α mAb treatment had no effect on ALI-associated hypoxemia, while it showed a trend toward a reduction in albumin leakage (Figures 1F–J). The reason for these differences observed between the *Il1*α^−/−^ mice and the use of anti-IL-1α mAb treatment is unknown, although IL-1α has been proposed to have intracellular effects separate from its secretion and signaling as a cytokine (42–45). Unlike anti-IL-1α mAb, intratracheal anti-IL-1β mAb treatment significantly improved arterial oxygenation as well as albumin level in BALF (Figures 1K–O). Importantly, IL-1β level in BALF from *Il1*α^−/−^ mice or anti-IL-1α mAb-treated mice did not change compared with control mice (Figures 1E,J). Moreover, IL-1α level in BALF from anti-IL-1β-mAb treated mice was significantly lower than that from control mice (Figure 1O). These findings demonstrate that in this experimental “two-hit” ALI model, developing hypoxemia predominanatly depends on IL-1β rather than IL-1α.

### Myeloid Cell-Specific Autophagy Deficient Mice Develop Severe Hypoxemia in LPS/MV-Induced ALI With Increased IL-1β

**Figure 2:**
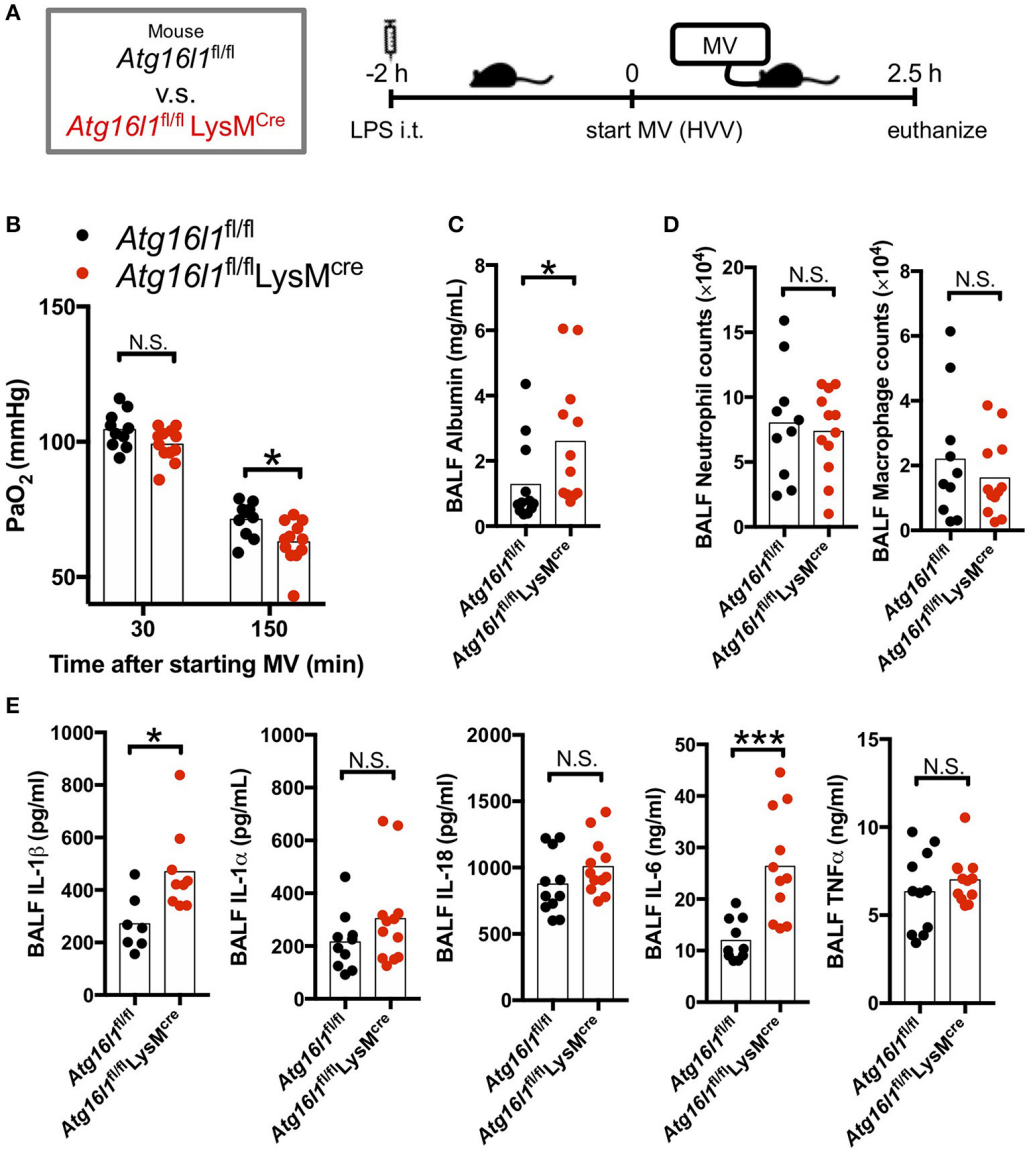
Myeloid cell-specific autophagy deficient mice developed worse lung permeability and oxygenation. **(A)** Study protocol. Two hours before starting mechanical ventilation (MV), 0.2 mg/kg of intratracheal (i.t.) LPS was administered to *Atg16l1*^fl/fl^ mice and to *Atg16l1*^fl/fl^ *LysM*^cre^ mice. Mice were then anesthetized and placed on high volume mechanical ventilation (HVV) with tidal volumes of 30 ml/kg with 3 cmH_2_O positive end-expiratory pressure (PEEP) for 2.5 h. **(B)** The effect of conditional knockout of *Atg16l1* on arterial partial pressure of oxygen (PaO_2_). PaO_2_ was measured at 30 and 150 min after starting MV. *indicates significant difference (adjusted *P* < 0.05), determined by two-way ANOVA followed by Sidak's multiple comparisons test. **(C)** Lung permeability determined by albumin in BALF. **(D)** Absolute counts of neutrophils and macrophages in BALF. **(E)** Cytokine levels determined in BALF. **(C–E)** *,***indicates *P* < 0.05, and *P* < 0.001, respectively, determined by Mann-Whitney *U-*test. N.S., not significant.

Autophagy removes damaged mitochondria generated by reactive oxygen species (ROS) and suppresses inflammasome-mediated IL-1β/IL-18 production (46). Thus, we hypothesized that inhibition of autophagy would enhance the production of IL-1β by AMs and therefore aggravate the lung injury in LPS/MV-induced ALI mice model. To test the role of myeloid specific autophagy deficiency, we induced ALI with the LPS + MV model (Figure 2A) in the *Atg16l1*^fl/fl^ *LysM*^Cre^ mice, whose loxP-flanked *Atg16l1* gene exon 3 were deleted in *LysM*^cre^ expressing cells including macrophages (32, 33). *Atg16l1*^fl/fl^ *LysM*^*Cre*^ mice developed more severe hypoxemia compared with *Atg16l1*^fl/fl^ littermate control mice (Figure 2B), as well as a significant increase in BALF albumin (Figure 2C), indicating increased lung vascular leakage. However, while we observed increased lung permeability and increased hypoxemia in the *Atg16l1*^fl/fl^ *LysM*^*Cre*^ mice, we did not observe any significant changes in airway neutrophil or macrophage numbers (Figure 2D), nor any difference in gross pathology of inflammation in the lungs compared with littermate controls (Figure S2). We did observe significantly increased amounts of IL-1β and IL-6 in BALF in *Atg16l1*^fl/fl^ *LysM*^*Cre*^ mice compared with *Atg16l1*^fl/fl^ control mice, which supports the key role of IL-1β in this model (Figure 2E). Since IL-6 and TNF-α production was not enhanced (Figures S3B,C) while IL-1β production was increased (Figure S3A) in *Atg16l1*^fl/fl^*LysM*^*cre*^ macrophages *in vitro*, the increase of IL-6 in BALF most likely represents down-stream responses to the increased IL-1β in *Atg16l1*^fl/fl^*LysM*^*cre*^ mice. Moreover, although we observed a tendency for increased IL-1α and IL-18 in *Atg16l1*^fl/fl^ *LysM*^*Cre*^ mice, we did not find any statistical significant differences for IL-1α, TNF-α, and IL-18 levels in BALF (Figure 2E). These results suggest that autophagy plays an important regulatory role in myeloid cells in proinflammatory cytokines secretion, primarily IL-1β, lung vascular leakage, and subsequent hypoxemia during LPS/MV-induced lung injury.

### Fasting Attenuates Hypoxemia in LPS/MV-Induced ALI With Decreased IL-1β Level

**Figure 3:**
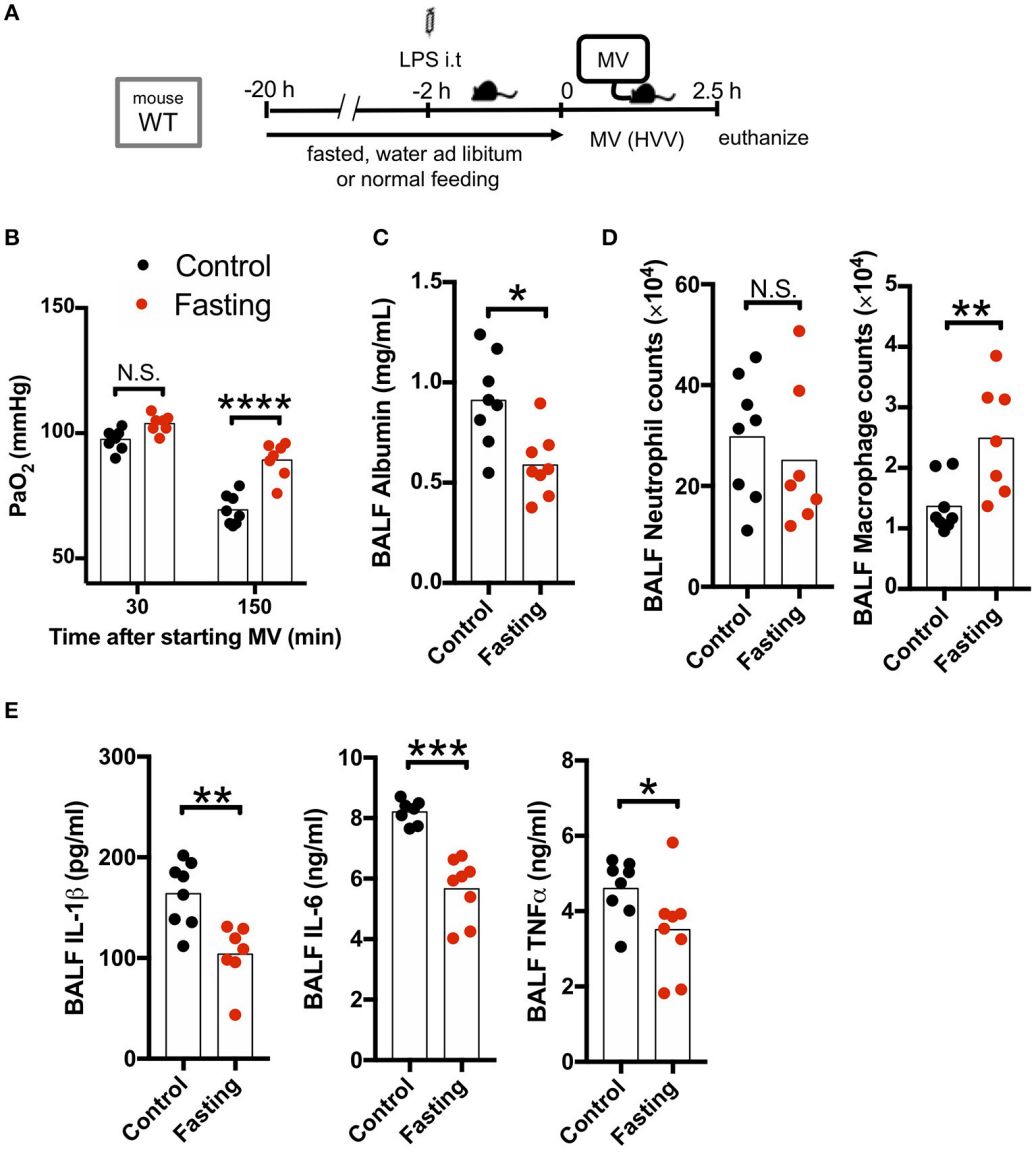
Fasting rescues two-hit acute lung injury. **(A)** Study protocol. Before starting mechanical ventilation (MV), one group of wild type (WT) mice were fasted with free-access of water, while the other was fed *ad libitum*. Two hours before starting MV, 0.2 mg/kg i.t. LPS was administered. Then, mice were anesthetized and placed on high volume mechanical ventilation (HVV) with tidal volumes of 30 ml/kg with 3 cmH_2_O positive end-expiratory pressure (PEEP) for 2.5 h. **(B)** The effect of starvation on arterial partial pressure of oxygen (PaO_2_). PaO_2_ was measured at 30 and 150 min after starting MV. ^****^indicates significant difference (adjusted *P* < 0.0001), determined by two-way ANOVA followed by Sidak's multiple comparisons test. **(C)** Lung permeability determined by albumin in BALF. **(D)** Absolute counts of neutrophils and macrophages in BALF. **(E)** Cytokine levels determined in BALF. **(C–E)** *,**,***indicates *P* < 0.05, *P* < 0.01, and *P* < 0.001, respectively, determined by Mann-Whitney *U-*test. N.S., not significant.

As our LPS plus MV induced “two-hit” ALI model was IL-1β dependent, and autophagy deficiency in macrophages led to worsened hypoxemia and increased IL-1β production, we hypothesized that induction of autophagy would be beneficial in this experimental mouse model. We first employed fasting, a well-accepted model to induce autophagy. Fasting and starvation induce a rapid and strong induction of autophagy in mice (38) and 24 h fasting induces autophagy in the lung and ameliorates lung inflammation (39). We also confirmed that our fasting protocol induced autophagy in the lung (Figure S4). We therefore fasted WT mice for 20 h before starting MV and found a dramatic attenuation in the development of hypoxemia (Figures 3A,B), as well as a significant decrease in albumin, IL-1β, IL-6, and TNFα in BALF (Figures 3C,E) following the LPS plus MV injury model. Moreover, while neutrophil infiltration into alveolar space was not affected by fasting, AM numbers were significantly increased (Figure 3D). Thus, fasting-induced autophagy inhibited IL-1β release and suppressed LPS/MV-induced lung permeability as well as lung BALF cytokine levels, resulting in improved arterial oxygenation.

### Trehalose, an mTOR-Independent Autophagy Enhancer, Does Not Attenuate Hypoxemia in LPS/MV-Induced ALI

**Figure 4:**
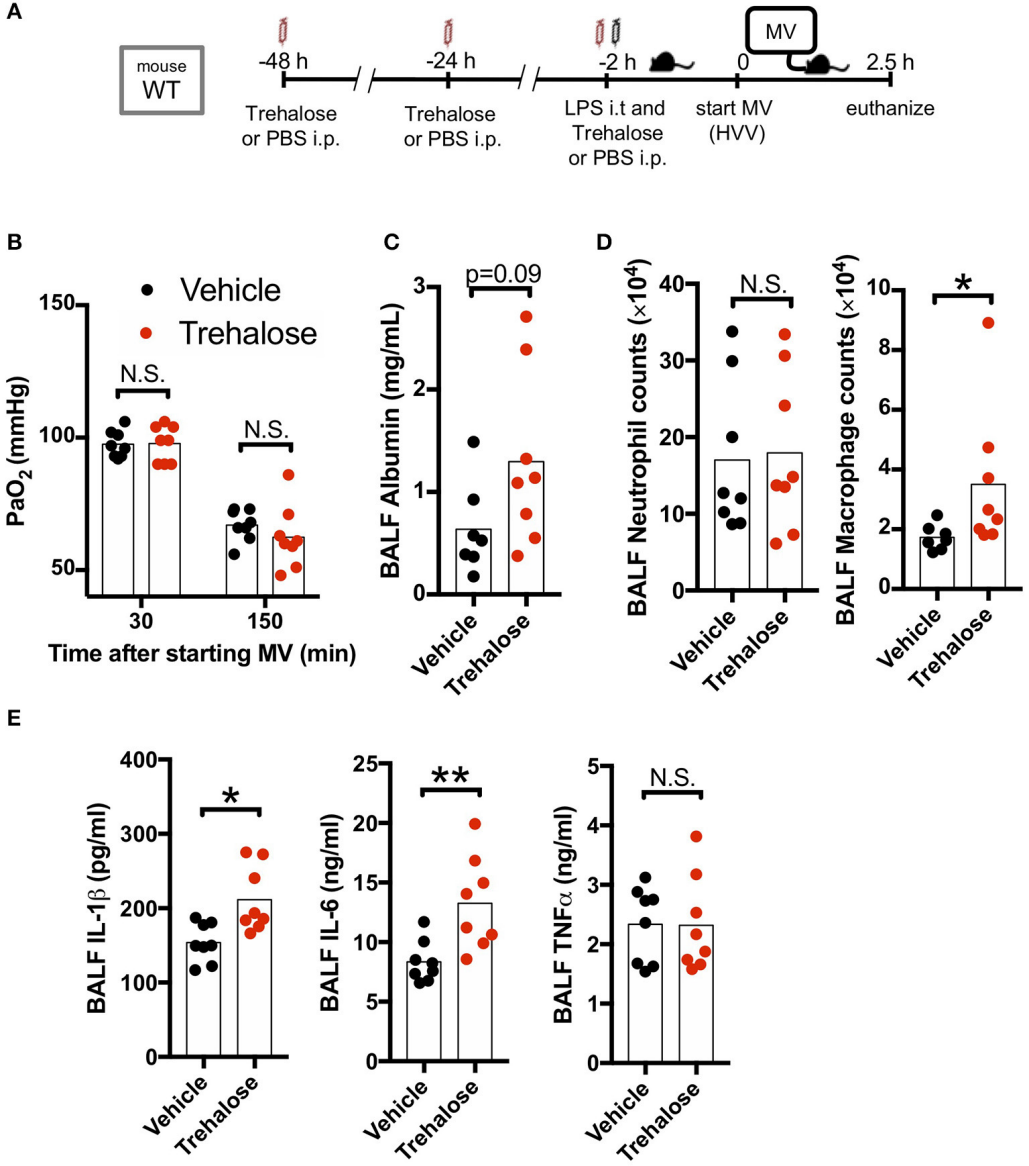
Trehalose treatment does not rescue two-hit acute lung injury. **(A)** Study protocol. Forty eight, twenty four, and two hours before starting mechanical ventilation (MV), 1 g/kg of intraperitoneal trehalose or PBS was administered. Two hours before starting MV, 0.2 mg/kg i.t. LPS was administered. Then, mice were anesthetized and placed on high volume mechanical ventilation (HVV) with tidal volumes of 30 ml/kg with 3 cmH_2_O positive end-expiratory pressure (PEEP) for 2.5 h. **(B)** The effect of trehalose on arterial partial pressure of oxygen (PaO_2_). PaO_2_ was measured at 30 and 150 min after starting MV. No significance was detected in PaO_2_ between vehicle and trehalose groups by two-way ANOVA followed by Sidak's multiple comparisons test. **(C)** Lung permeability determined by albumin in BALF. **(D)** Absolute counts of neutrophils and macrophages in BALF. **(E)** Cytokine levels determined in BALF. **(C–E)** *,**indicates *P* < 0.05 and *P* < 0.01, respectively, determined by Mann-Whitney *U-*test. N.S., not significant.

We next hypothesized that use of another known autophagy inducer would also decrease the production of IL-1β by AMs and therefore provide a potential protection against hypoxemia in this experimental ALI model. Trehalose, a naturally occurring disaccharide, has been proposed as an mTOR-independent autophagy enhancer (47) and was reported to have therapeutic effects against atherosclerosis, myocardial infarction, spinal cord injury, traumatic brain injury, and Alzheimer's disease (40, 48–51). Therefore, we investigated the therapeutic effect of this mTOR-independent autophagy activator in our two-hit ALI mouse model. However, we observed that trehalose treatment had no protective effect on arterial oxygenation and instead had increased BALF albumin (increased permeability), increased IL-1β, and IL-6 (Figures 4A–C,E) although we confirmed that our trehalose treatment induced autophagy in the lung (Figure S4). Additionally, while there was no difference in neutrophil infiltration, trehalose treatment did lead to increased AMs in BALF (Figure 4D). These data indicated that contrary to fasting induced mTOR-dependent autophagy, the mTOR-independent autophagy activation by trehalose was either not effective in inhibiting inflammasome activation and IL-1β release, or instead had a detrimental off-target effect.

## Discussion

It is recognized that NLRP3 inflammasome activation in AM mediates lung inflammatory responses during MV (13). The role of autophagy in ALI, including VILI, has been controversial with some studies suggesting a protective and others a detrimental role of autophagy (17, 18). In this study we found that autophagy in myeloid cells plays a protective role in the experimental LPS/MV-induced “two-hit” ALI model. Mice lacking the myeloid cell-specific *Atg16l1*, an essential autophagy gene, developed significantly increased lung permeability and more severe hypoxemia in this LPS/MV-induced ALI model, and this aggravation was related to increased IL-1β production in alveolar space. Supporting the protective role of autophagy in this LPS plus MV model, we also observed that induction of autophagy by fasting attenuated lung permeability and hypoxemia. Importantly, the severity of lung injury and hypoxemia were associated with the level of IL-1β release into alveolar space, which is dependent on inflammasome activation in AMs.

ATG16L1 is a critical component of a complex that lipidates LC3, the ubiquitin-like molecule, to enhance autophagosome formation. Genetic loss of this protein in macrophages was shown to enhance inflammasome activation and production of IL-1β *in vitro* by LPS plus ATP or nigericin (28–31). Indeed, using myeloid specific *Atg16l1* autophagy KO mice, we found that IL-1β production in alveolar space was increased during the LPS plus MV induced “two-hit” ALI model. Additionally, we found that fasting, a powerful inducer of autophagy, led to decreased intraalveolar IL-1β production and better oxygenation of the mice during LPS/MV-induced ALI. While fasting led to an increase in AM numbers, it inhibited IL-1β production and led to a reduction in lung permeability in two-hit ALI model. Fasting activates autophagy by hindering mammalian target of rapamycin (mTOR) (52). Shi et al. clearly demonstrated that augmenting autophagy in THP-1 cells by amino acid starvation *in vitro* attenuates inflammasome activity, resulting in less IL-1β production after treatment with LPS plus poly(dA-dT) or nigericin (53). Although we employed fasting as a reliable and established method to enhance systemic autophagy by starvation, we did not evaluate the other systemic metabolic effects of starvation. Indeed, we observed that 20 h fasting also inhibited the TNF-α as well as IL-1β levels in BALF in this two-hit ALI model, while *Atg16l1*^fl/fl^ *LysM*^Cre^ did not alter BALF TNF-α concentrations. This raises the possibility that decreased IL-1β level in BALF in the fasting group might be also affected by regulation of transcription as well as regulation of NLRP3 inflammasome activation. Indeed, starvation can lead to reduced TNF-α and IL-1β transcription (29, 30). On the other hand, *Atg5* KO macrophages increased M1 polarization and produced more TNF-α, IL-6 at both mRNA and protein levels under the M1 polarization conditions (12 h LPS + IFN-γ) (54). However, we observed no changes in the TNF-α levels in the BALF from *Atg16l1*^fl/fl^ *LysM*^*Cre*^ mice during LPS+MV, indicating that the nitric oxide pathway, a master regulator of M1 polarization, may not be induced or may not be regulated by autophagy in this short time window used in our experimental model. Thus, LPS-stimulated inflammasome-independent pro-inflammatory cytokines may not be altered by autophagy in macrophages, or reduced correlatedly in autophagy deficient macrophages by cell death (33).

Cellular stress, its dampening by autophagy, and apoptosis are highly interregulated as part of normal cellular homeostasis. Thus, autophagy and inflammasome activation occur nearly simultaneously (28, 55). Therefore, the interrelationship of these various pathways can lead to inconclusive or confounding interpretations depending on the experimental models used (56). Dupont et al. previously demonstrated that starvation induces more NLRP3 inflammasome activation and IL-1β secretion in BMDM (57), however, they treated LPS-primed BMDM with starvation buffer (EBSS) for only 1 h with NLRP3 inflammasome activators, which is an extremely short duration to induce starvation. Zhang et al. reported that high tidal MV activated autophagy in AMs, but this resulting in aggravated inflammatory responses (17). They found that inhibition of autophagy by systemic 3-MA treatment, and *Atg5* silenced BMDM adaptive transfer to clodronate-treated mice, significantly attenuated activation of the NLRP3 inflammasome during only MV-induced ALI (17). In our study we have demonstrated the opposite role of autophagy in IL-1β production and protection of inflammation, injury and hypoxemia in LPS plus MV-induced ALI. Our study used LPS plus MV as a “two-hit” model, while the study by Zhang et al. was only for MV (17). Indeed, whether autophagy is protective or detrimental in lung inflammation and injury likely depends on the specific model used and thus specific stimuli, on the extent of its activation, and the specific cell type (17, 18, 58). Nevertheless, most studies agree that autophagy negatively regulates NLRP3 inflammasome activation (15, 59). Multiple previous studies demonstrated that high volume MV or mechanical stretch enhances the effect of TLR4 agonist (e.g., LPS) on lung cells such as AMs (19, 60–63). It is also established that autophagy attenuates TLR4-dependent IL-1β production (15). TIR-domain-containing inducing interferon-β (TRIF)-mediated ROS production is greatly aggravated in autophagy-deficient macrophages, which then leads to higher NLRP3 inflammasome activation and IL-1β release. LPS-induced ALI is exacerbated by inhibiting autophagy (64), and treatment with rapamycin, a major autophagy inducer, attenuates LPS-induced IL-1β secretion from macrophages (29). Thus, our findings agree with these previous studies and indicate that MV promotes the sepsis-activated NLRP3 inflammasome, which is negatively regulated by autophagy.

Interestingly, we observed dissociative results beween IL-1β and IL-18, both of which are inflammasome-dependent cytokines, in our “two-hit” model using *Atg16l1*^fl/fl^ *LysM*^*Cre*^ mice (i.e., significantly increased IL-1β but no IL-18 difference in *Atg16l1*^fl/fl^ *LysM*^*Cre*^ mice). A possible explanation for the dissociation between these cytokines can be the unique IL-18 production in lung epithelial cells. A previous study (65) demonstrated that epithelial cells constitutively express pro-IL-18 without additional stimuli, and that mature IL-18 is released through a caspase-1-dependent pathway without priming. We have demonstrated that (1) *Casp1*^−/−^ mice in our ALI model were rescued from developing hypoxemia along with significantly low amounts of IL-18 as well as IL-1β in BAL (Figures S5A,B); (2) a high volume ventilation (HVV) “single-hit” model can also produce equal amounts of IL-18 as the LPS plus HVV “two-hit” model (Figures S5C,D), while the production of IL-1β requires the two-hit (12). These data indicate that IL-18 in our VILI model is likely derived from epithelial cells through caspase-1-dependent manner. Therefore, as autophagy function in epithelial cells is intact in *Atg16l1*^fl/fl^ *LysM*^Cre^ mice, overall IL-18 secretion was not altered in myeloid cell-*Atg16l1* KO mice.

We observed that trehalose treatment aggravated ALI with increased intra-alveolar IL-1β, which was contrary to our expectation. Trehalose has received attention as an mTOR-independent autophagy activator (47). Mice treated with trehalose (2% in the drinking water for 6 weeks or aerosolized trehalose administration for 24 h) prior to chlorine exposure demonstrated increased bioenergetic function and attenuated lung inflammation (66). Likewise, trehalose blunted LPS-induced ALI (41). In contrast, however, there is still controversy surrounding the role of trehalose as an autophagy inducer (67). Some *in vitro* studies demonstrated that trehalose treatment inhibits autophagic flux from autophagosome to autolysosome (68). Lee et al. proposed an indirect mechanism of trehalose on its neuroprotective effects through regulating gut microbiota (68). Therefore, whether trehalose is protective or harmful in lung injury models may depend on the dose and route of administration, or the extent of injurious burden of the model.

We have previously reported that in the LPS plus MV-induced “two-hit” model developed hypoxemia in a NLRP3 inflammasome dependent manner, and was attenuated by the IL-1R antagonist, anakinra, which inhibits both IL-1α and IL-1β (12). Therefore, in the current study we determined the relative role of IL-1α vs. IL-1β in this ALI model. We found that IL-1β but not IL-1α is critical in the development of the LPS plus MV-induced “two-hit” model of ALI. Our data showed that IL-1α release into alveolar space was significantly inhibited by intratracheal anti-IL-1β mAb treatment whereas IL-1β release into alveolar space was not suppressed in *Il1*α^−/−^ mice, or by anti-IL-1α mAb treatment. These results indicate the predominant role of IL-1β in the development of LPS/MV-induced hypoxemia.

While this study shows the importance of autophagy in myeloid cells in our two-hit model of ALI, there are a few limitations as a result of our approach. First, we have only focused on the functional parameters of the lung (PaO_2_) and the inflammation (e.g., cytokines, cell counts) as readouts for our study. Systemic metabolic effects by these interventions should be evaluated in the future studies. Second, while we demonstrated the crucial involvement of IL-1β in the development of VILI/ALI by treating mice with anti-IL-1β mAb at the same point as LPS administration, we did not test the effect of late administration of anti-IL-1β mAb (i.e., anti-IL-1β antibody administration after LPS treatment) from a translational point of view. However, since the onset of VILI is predictable, and early mediators such as IL-1β and TNF-α can be modulated preventively (69, 70), prophylactic treatment against VILI may be the clue for a successful approach. In this context, a cytokine-targeting strategy, including anti-IL-1β, may be promising in the management of VILI, although a number of clinical trials in (predominantly sepsis) patients targeting a specific cytokine failed to show the effectiveness (69).

In summary, our data demonstrated that autophagy in myeloid cells plays a protective role in the development of LPS/MV-induced ALI through modifying IL-1β production. Although trehalose, a proposed mTOR-independent autophagy inducer, did not improve LPS/MV-induced ALI, fasting induced mTOR-dependent autophagy led to significantly improved arterial oxygenation and lung permeability, through significantly suppressed IL-1β production. Our observations confirmed that induction of mTOR-dependent autophagy and inhibition of IL-1β production, or the subsequent IL-1R signaling may be a promising treatment targets for LPS plus MV-induced ARDS and ALI. Further studies are required to better understand the mechanisms involved between mTOR-dependent and mTOR-independent autophagy inducers to thoroughly comprehend how they modulate lung injury in various experimental models.

## Data Availability Statement

All datasets generated for this study are included in the article/Supplementary Material.

## Ethics Statement

All experiments were performed according to Cedars-Sinai Medical Center Institutional Animal Care and Use Committee (IACUC) guidelines.

## Author Contributions

KS conceived and led the project. NN performed all of the experiments and wrote the manuscript. DMa and DMo assisted experiments. TC, MA, and KS provided critical editing and content to the manuscript as well as experimental design. KS supervised the study. All of the authors read and approved the final manuscript.

### Conflict of Interest

The authors declare that the research was conducted in the absence of any commercial or financial relationships that could be construed as a potential conflict of interest.

## References

[1] Force ADT Ranieri VM Rubenfeld GD Thompson BT Ferguson ND Caldwell E Acute respiratory distress syndrome: the Berlin Definition. JAMA. (2012) 307:2526–33. 10.1001/jama.2012.5669 22797452

[2] Matthay MA Zemans RL Zimmerman GA Arabi YM Beitler JR Mercat A Acute respiratory distress syndrome. Nat Rev Dis Primers. (2019) 5:18 10.1038/s41572-019-0069-0 30872586 PMC6709677

[3] Del Sorbo L Goligher EC McAuley DF Rubenfeld GD Brochard LJ Gattinoni L. Mechanical ventilation in adults with acute respiratory distress syndrome. Summary of the experimental evidence for the clinical practice guideline. Ann Am Thorac Soc. (2017) 14:S261–70. 10.1513/AnnalsATS.201704-345OT 28985479

[4] Rittayamai N Brochard L. Recent advances in mechanical ventilation in patients with acute respiratory distress syndrome. Eur Respir Rev. (2015) 24:132–40. 10.1183/09059180.00012414 25726563 PMC9487769

[5] Ganter MT Roux J Miyazawa B Howard M Frank JA Su G. Interleukin-1beta causes acute lung injury via alphavbeta5 and alphavbeta6 integrin-dependent mechanisms. Circ Res. (2008) 102:804–12. 10.1161/CIRCRESAHA.107.161067 18276918 PMC2739091

[6] Jacobs RF Tabor DR Burks AW Campbell GD. Elevated interleukin-1 release by human alveolar macrophages during the adult respiratory distress syndrome. Am Rev Respir Dis. (1989) 140:1686–92. 10.1164/ajrccm/140.6.1686 2604296

[7] Eyal FG Hamm CR Parker JC. Reduction in alveolar macrophages attenuates acute ventilator induced lung injury in rats. Intensive Care Med. (2007) 33:1212–8. 10.1007/s00134-007-0651-x 17468847

[8] Lindauer ML Wong J Iwakura Y Magun BE. Pulmonary inflammation triggered by ricin toxin requires macrophages and IL-1 signaling. J Immunol. (2009) 183:1419–26. 10.4049/jimmunol.0901119 19561099 PMC4467824

[9] Swanson KV Deng M Ting JP. The NLRP3 inflammasome: molecular activation and regulation to therapeutics. Nat Rev Immunol. (2019) 19:477–89. 10.1038/s41577-019-0165-0 31036962 PMC7807242

[10] Frank JA Pittet JF Wray C Matthay MA. Protection from experimental ventilator-induced acute lung injury by IL-1 receptor blockade. Thorax. (2008) 63:147–53. 10.1136/thx.2007.079608 17901159

[11] Kuipers MT Aslami H Janczy JR van der Sluijs KF Vlaar AP Wolthuis EK. Ventilator-induced lung injury is mediated by the NLRP3 inflammasome. Anesthesiology. (2012) 116:1104–15. 10.1097/ALN.0b013e3182518bc0 22531249

[12] Jones HD Crother TR Gonzalez-Villalobos RA Jupelli M Chen S Dagvadorj J. The NLRP3 inflammasome is required for the development of hypoxemia in LPS/mechanical ventilation acute lung injury. Am J Respir Cell Mol Biol. (2014) 50:270–80. 10.1165/rcmb.2013-0087OC 24007300 PMC3930947

[13] Wu J Yan Z Schwartz DE Yu J Malik AB Hu G. Activation of NLRP3 inflammasome in alveolar macrophages contributes to mechanical stretch-induced lung inflammation and injury. J Immunol. (2013) 190:3590–9. 10.4049/jimmunol.1200860 23436933 PMC3608749

[14] Mizumura K Cloonan S Choi ME Hashimoto S Nakahira K Ryter SW Autophagy: friend or foe in lung disease? Ann Am Thorac Soc. (2016) 13(Suppl 1):S40–7. 10.1513/AnnalsATS.201507-450MG 27027951 PMC5466160

[15] Saitoh T Akira S. Regulation of inflammasomes by autophagy. J Allergy Clin Immunol. (2016) 138:28–36. 10.1016/j.jaci.2016.05.009 27373323

[16] Lopez-Alonso I Aguirre A Gonzalez-Lopez A Fernandez AF Amado-Rodriguez L Astudillo A. Impairment of autophagy decreases ventilator-induced lung injury by blockade of the NF-kappaB pathway. Am J Physiol Lung Cell Mol Physiol. (2013) 304:L844–52. 10.1152/ajplung.00422.2012 23585228

[17] Zhang Y Liu G Dull RO Schwartz DE Hu G. Autophagy in pulmonary macrophages mediates lung inflammatory injury via NLRP3 inflammasome activation during mechanical ventilation. Am J Physiol Lung Cell Mol Physiol. (2014) 307:L173–85. 10.1152/ajplung.00083.2014 24838752 PMC4101793

[18] Mizumura K Cloonan SM Haspel JA Choi AMK. The emerging importance of autophagy in pulmonary diseases. Chest. (2012) 142:1289–99. 10.1378/chest.12-0809 23131937 PMC3494477

[19] Moriyama K Ishizaka A Nakamura M Kubo H Kotani T Yamamoto S. Enhancement of the endotoxin recognition pathway by ventilation with a large tidal volume in rabbits. Am J Physiol Lung Cell Mol Physiol. (2004) 286:L1114–21. 10.1152/ajplung.00296.2003 14633514

[20] Crimi E Zhang H Han RN Del Sorbo L Ranieri VM Slutsky AS. Ischemia and reperfusion increases susceptibility to ventilator-induced lung injury in rats. Am J Respir Crit Care Med. (2006) 174:178–86. 10.1164/rccm.200507-1178OC 16645175

[21] Gharib SA Liles WC Matute-Bello G Glenny RW Martin TR Altemeier WA. Computational identification of key biological modules and transcription factors in acute lung injury. Am J Respir Crit Care Med. (2006) 173:653–8. 10.1164/rccm.200509-1473OC 16387799

[22] Dhanireddy S Altemeier WA Matute-Bello G O'Mahony DS Glenny RW Martin TR. Mechanical ventilation induces inflammation, lung injury, and extra-pulmonary organ dysfunction in experimental pneumonia. Lab Invest. (2006) 86:790–9. 10.1038/labinvest.3700440 16855596

[23] Szczepaniak WS Zhang Y Hagerty S Crow MT Kesari P Garcia JG. Sphingosine 1-phosphate rescues canine LPS-induced acute lung injury and alters systemic inflammatory cytokine production *in vivo*. Transl Res. (2008) 152:213–24. 10.1016/j.trsl.2008.09.002 19010292 PMC2605585

[24] Dixon DL De Smet HR Bersten AD. Lung mechanics are both dose and tidal volume dependant in LPS-induced lung injury. Respir Physiol Neurobiol. (2009) 167:333–40. 10.1016/j.resp.2009.06.008 19539791

[25] Charles PE Tissieres P Barbar SD Croisier D Dufour J Dunn-Siegrist I. Mild-stretch mechanical ventilation upregulates toll-like receptor 2 and sensitizes the lung to bacterial lipopeptide. Crit Care. (2011) 15:R181. 10.1186/cc10330 21794115 PMC3387624

[26] Wosten-van Asperen RM Lutter R Specht PA van Woensel JB van der Loos CM Florquin S. Ventilator-induced inflammatory response in lipopolysaccharide-exposed rat lung is mediated by angiotensin-converting enzyme. Am J Pathol. (2010) 176:2219–27. 10.2353/ajpath.2010.090565 20304959 PMC2861087

[27] Jia X Malhotra A Saeed M Mark RG Talmor D. Risk factors for ARDS in patients receiving mechanical ventilation for > 48 h. Chest. (2008) 133:853–61. 10.1378/chest.07-1121 18263691 PMC2628459

[28] Nakahira K Haspel JA Rathinam VA Lee SJ Dolinay T Lam HC. Autophagy proteins regulate innate immune responses by inhibiting the release of mitochondrial DNA mediated by the NALP3 inflammasome. Nat Immunol. (2011) 12:222–30. 10.1038/ni.1980 21151103 PMC3079381

[29] Harris J Hartman M Roche C Zeng SG O'Shea A Sharp FA. Autophagy controls IL-1beta secretion by targeting pro-IL-1beta for degradation. J Biol Chem. (2011) 286:9587–97. 10.1074/jbc.M110.202911 21228274 PMC3058966

[30] Crisan TO Plantinga TS van de Veerdonk FL Farcas MF Stoffels M Kullberg BJ. Inflammasome-independent modulation of cytokine response by autophagy in human cells. PLoS ONE. (2011) 6:e18666. 10.1371/journal.pone.0018666 21490934 PMC3072416

[31] Domiciano TP Wakita D Jones HD Crother TR Verri WA Jr Arditi M. Quercetin inhibits inflammasome activation by interfering with ASC oligomerization and prevents interleukin-1 mediated mouse vasculitis. Sci Rep. (2017) 7:41539. 10.1038/srep41539 28148962 PMC5288648

[32] Zhang H Zheng L McGovern DP Hamill AM Ichikawa R Kanazawa Y. Myeloid ATG16L1 facilitates host-bacteria interactions in maintaining intestinal homeostasis. J Immunol. (2017) 198:2133–46. 10.4049/jimmunol.1601293 28130498 PMC5322190

[33] Crother TR Porritt RA Dagvadorj J Tumurkhuu G Slepenkin AV Peterson EM. Autophagy limits inflammasome during chlamydia pneumoniae infection. Front Immunol. (2019) 10:754. 10.3389/fimmu.2019.00754 31031755 PMC6473188

[34] Horai R Asano M Sudo K Kanuka H Suzuki M Nishihara M. Production of mice deficient in genes for interleukin (IL)-1alpha, IL-1beta, IL-1alpha/beta, and IL-1 receptor antagonist shows that IL-1beta is crucial in turpentine-induced fever development and glucocorticoid secretion. J Exp Med. (1998) 187:1463–75. 10.1084/jem.187.9.1463 9565638 PMC2212263

[35] Nosaka N Crother TR Chen S Arditi M Shimada K. Optimal tube length of orotracheal intubation for mice. Lab Anim. (2019) 53:79–83. 10.1177/0023677218765032 29649932 PMC6203655

[36] Provoost S Maes T Pauwels NS Vanden Berghe T Vandenabeele P Lambrecht BN. NLRP3/caspase-1-independent IL-1beta production mediates diesel exhaust particle-induced pulmonary inflammation. J Immunol. (2011) 187:3331–7. 10.4049/jimmunol.1004062 21844393

[37] Pauwels NS Bracke KR Dupont LL Van Pottelberge GR Provoost S Vanden Berghe T. Role of IL-1alpha and the Nlrp3/caspase-1/IL-1beta axis in cigarette smoke-induced pulmonary inflammation and COPD. Eur Respir J. (2011) 38:1019–28. 10.1183/09031936.00158110 21622588

[38] Moulis M Vindis C. Methods for measuring autophagy in mice. Cells. (2017) 6:E14. 10.3390/cells6020014 28594368 PMC5492018

[39] Aguirre A Lopez-Alonso I Gonzalez-Lopez A Amado-Rodriguez L Batalla-Solis E Astudillo A. Defective autophagy impairs ATF3 activity and worsens lung injury during endotoxemia. J Mol Med. (2014) 92:665–76. 10.1007/s00109-014-1132-7 24535031

[40] Sciarretta S Yee D Nagarajan N Bianchi F Saito T Valenti V. Trehalose-induced activation of autophagy improves cardiac remodeling after myocardial infarction. J Am Coll Cardiol. (2018) 71:1999–2010. 10.1016/j.jacc.2018.02.066 29724354 PMC6347412

[41] Minutoli L Altavilla D Bitto A Polito F Bellocco E Lagana G. Trehalose: a biophysics approach to modulate the inflammatory response during endotoxic shock. Eur J Pharmacol. (2008) 589:272–80. 10.1016/j.ejphar.2008.04.005 18555988

[42] Stevenson FT Turck J Locksley RM Lovett DH. The N-terminal propiece of interleukin 1 alpha is a transforming nuclear oncoprotein. Proc Natl Acad Sci USA. (1997) 94:508–13. 10.1073/pnas.94.2.508 9012814 PMC19543

[43] Buryskova M Pospisek M Grothey A Simmet T Burysek L. Intracellular interleukin-1alpha functionally interacts with histone acetyltransferase complexes. J Biol Chem. (2004) 279:4017–26. 10.1074/jbc.M306342200 14612453

[44] Werman A Werman-Venkert R White R Lee JK Werman B Krelin Y. The precursor form of IL-1alpha is an intracrine proinflammatory activator of transcription. Proc Natl Acad Sci USA. (2004) 101:2434–9. 10.1073/pnas.0308705101 14983027 PMC356968

[45] Kawaguchi Y Nishimagi E Tochimoto A Kawamoto M Katsumata Y Soejima M. Intracellular IL-1alpha-binding proteins contribute to biological functions of endogenous IL-1alpha in systemic sclerosis fibroblasts. Proc Natl Acad Sci USA. (2006) 103:14501–6. 10.1073/pnas.0603545103 16971486 PMC1599989

[46] Zhou R Yazdi AS Menu P Tschopp J. A role for mitochondria in NLRP3 inflammasome activation. Nature. (2011) 469:221–5. 10.1038/nature09663 21124315

[47] Mardones P Rubinsztein DC Hetz C. Mystery solved: trehalose kickstarts autophagy by blocking glucose transport. Sci Signal. (2016) 9:fs2. 10.1126/scisignal.aaf1937 26905424

[48] Sergin I Evans TD Zhang X Bhattacharya S Stokes CJ Song E. Exploiting macrophage autophagy-lysosomal biogenesis as a therapy for atherosclerosis. Nat Commun. (2017) 8:15750. 10.1038/ncomms15750 28589926 PMC5467270

[49] Nazari-Robati M Akbari M Khaksari M Mirzaee M. Trehalose attenuates spinal cord injury through the regulation of oxidative stress, inflammation and GFAP expression in rats. J Spinal Cord Med. (2019) 42:387–394. 10.1080/10790268.2018.1527077 30513271 PMC6522923

[50] Portbury SD Hare DJ Finkelstein DI Adlard PA. Trehalose improves traumatic brain injury-induced cognitive impairment. PLoS ONE. (2017) 12:e0183683. 10.1371/journal.pone.0183683 28837626 PMC5570321

[51] Portbury SD Hare DJ Sgambelloni C Perronnes K Portbury AJ Finkelstein DI. Trehalose improves cognition in the transgenic Tg2576 mouse model of Alzheimer's disease. J Alzheimers Dis. (2017) 60:549–60. 10.3233/JAD-170322 28869469 PMC5611803

[52] He L Zhang J Zhao J Ma N Kim SW Qiao S. Autophagy: the last defense against cellular nutritional stress. Adv Nutr. (2018) 9:493–504. 10.1093/advances/nmy011 30032222 PMC6054220

[53] Shi CS Shenderov K Huang NN Kabat J Abu-Asab M Fitzgerald KA. Activation of autophagy by inflammatory signals limits IL-1beta production by targeting ubiquitinated inflammasomes for destruction. Nat Immunol. (2012) 13:255–63. 10.1038/ni.2215 22286270 PMC4116819

[54] Liu K Zhao E Ilyas G Lalazar G Lin Y Haseeb M. Impaired macrophage autophagy increases the immune response in obese mice by promoting proinflammatory macrophage polarization. Autophagy. (2015) 11:271–84. 10.1080/15548627.2015.1009787 25650776 PMC4502775

[55] Saitoh T Fujita N Jang MH Uematsu S Yang BG Satoh T. Loss of the autophagy protein Atg16L1 enhances endotoxin-induced IL-1beta production. Nature. (2008) 456:264–8. 10.1038/nature07383 18849965

[56] Pun NT Subedi A Kim MJ Park PH. Globular adiponectin causes tolerance to LPS-induced TNF-alpha expression via autophagy induction in RAW 264.7 macrophages: involvement of SIRT1/FoxO3A axis. PLoS ONE. (2015) 10:e0124636. 10.1371/journal.pone.0124636 25961287 PMC4427353

[57] Dupont N Jiang S Pilli M Ornatowski W Bhattacharya D Deretic V. Autophagy-based unconventional secretory pathway for extracellular delivery of IL-1beta. EMBO J. (2011) 30:4701–11. 10.1038/emboj.2011.398 22068051 PMC3243609

[58] Tanaka A Jin Y Lee SJ Zhang M Kim HP Stolz DB. Hyperoxia-induced LC3B interacts with the Fas apoptotic pathway in epithelial cell death. Am J Respir Cell Mol Biol. (2012) 46:507–14. 10.1165/rcmb.2009-0415OC 22095627 PMC3359946

[59] Takahama M Akira S Saitoh T. Autophagy limits activation of the inflammasomes. Immunol Rev. (2018) 281:62–73. 10.1111/imr.12613 29248000

[60] Pugin J Dunn I Jolliet P Tassaux D Magnenat JL Nicod LP. Activation of human macrophages by mechanical ventilation *in vitro*. Am J Physiol. (1998) 275:L1040–50. 10.1152/ajplung.1998.275.6.L1040 9843840

[61] Kuipers MT Vogl T Aslami H Jongsma G van den Berg E Vlaar AP. High levels of S100A8/A9 proteins aggravate ventilator-induced lung injury via TLR4 signaling. PLoS ONE. (2013) 8:e68694. 10.1371/journal.pone.0068694 23874727 PMC3715539

[62] Kuhn H Petzold K Hammerschmidt S Wirtz H. Interaction of cyclic mechanical stretch and toll-like receptor 4-mediated innate immunity in rat alveolar type II cells. Respirology. (2014) 19:67–73. 10.1111/resp.12149 23796194

[63] Dai H Pan L Lin F Ge W Li W He S. Mechanical ventilation modulates Toll-like receptors 2:4, and 9 on alveolar macrophages in a ventilator-induced lung injury model. J Thorac Dis. (2015) 7:616–24. 10.3978/j.issn.2072-1439.2015.02.10 25973227 PMC4419314

[64] Zhang D Zhou J Ye LC Li J Wu Z Li Y. Autophagy maintains the integrity of endothelial barrier in LPS-induced lung injury. J Cell Physiol. (2018) 233:688–98. 10.1002/jcp.25928 28328069

[65] Lu H Shen C Brunham RC. Chlamydia trachomatis infection of epithelial cells induces the activation of caspase-1 and release of mature IL-18. J Immunol. (2000) 165:1463–9. 10.4049/jimmunol.165.3.1463 10903751

[66] Jurkuvenaite A Benavides GA Komarova S Doran SF Johnson M Aggarwal S. Upregulation of autophagy decreases chlorine-induced mitochondrial injury and lung inflammation. Free Radic Biol Med. (2015) 85:83–94. 10.1016/j.freeradbiomed.2015.03.039 25881550 PMC4508227

[67] Yoon YS Cho ED Jung Ahn W Won Lee K Lee SJ Lee HJ. Is trehalose an autophagic inducer? Unraveling the roles of non-reducing disaccharides on autophagic flux and alpha-synuclein aggregation. Cell Death Dis. (2017) 8:e3091. 10.1038/cddis.2017.501 28981090 PMC5682667

[68] Lee HJ Yoon YS Lee SJ. Mechanism of neuroprotection by trehalose: controversy surrounding autophagy induction. Cell Death Dis. (2018) 9:712. 10.1038/s41419-018-0749-9 29907758 PMC6003909

[69] Wilson MR Takata M. Inflammatory mechanisms of ventilator-induced lung injury: a time to stop and think? Anaesthesia. (2013) 68:175–8. 10.1111/anae.12085 23173768

[70] dos Santos CC. The role of the inflammasome in ventilator-induced lung injury. Am J Respir Crit Care Med. (2012) 185:1141–4. 10.1164/rccm.201204-0649ED 22661520

