# Supplementary figures and images for: Autophagy Protects Against Developing Increased Lung Permeability and Hypoxemia by Down Regulating Inflammasome Activity and IL-1β in LPS Plus Mechanical Ventilation-Induced Acute Lung Injury
Source: Front Immunol. 2020 Feb 14;11:207. doi: 10.3389/fimmu.2020.00207 (PMC7033480; doi:10.3389/fimmu.2020.00207)

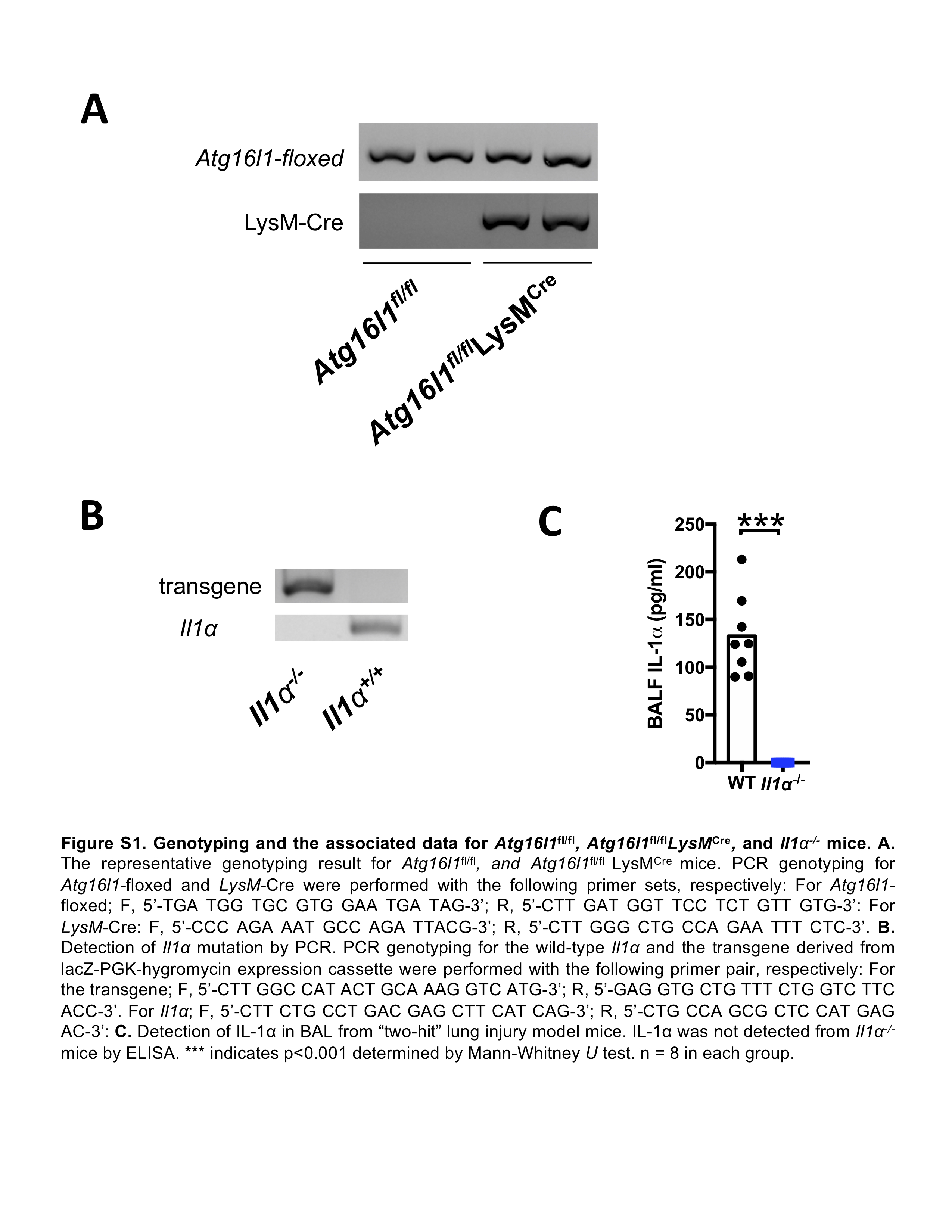

Supplement: Supplementary file 1 [file Image_1.jpg]

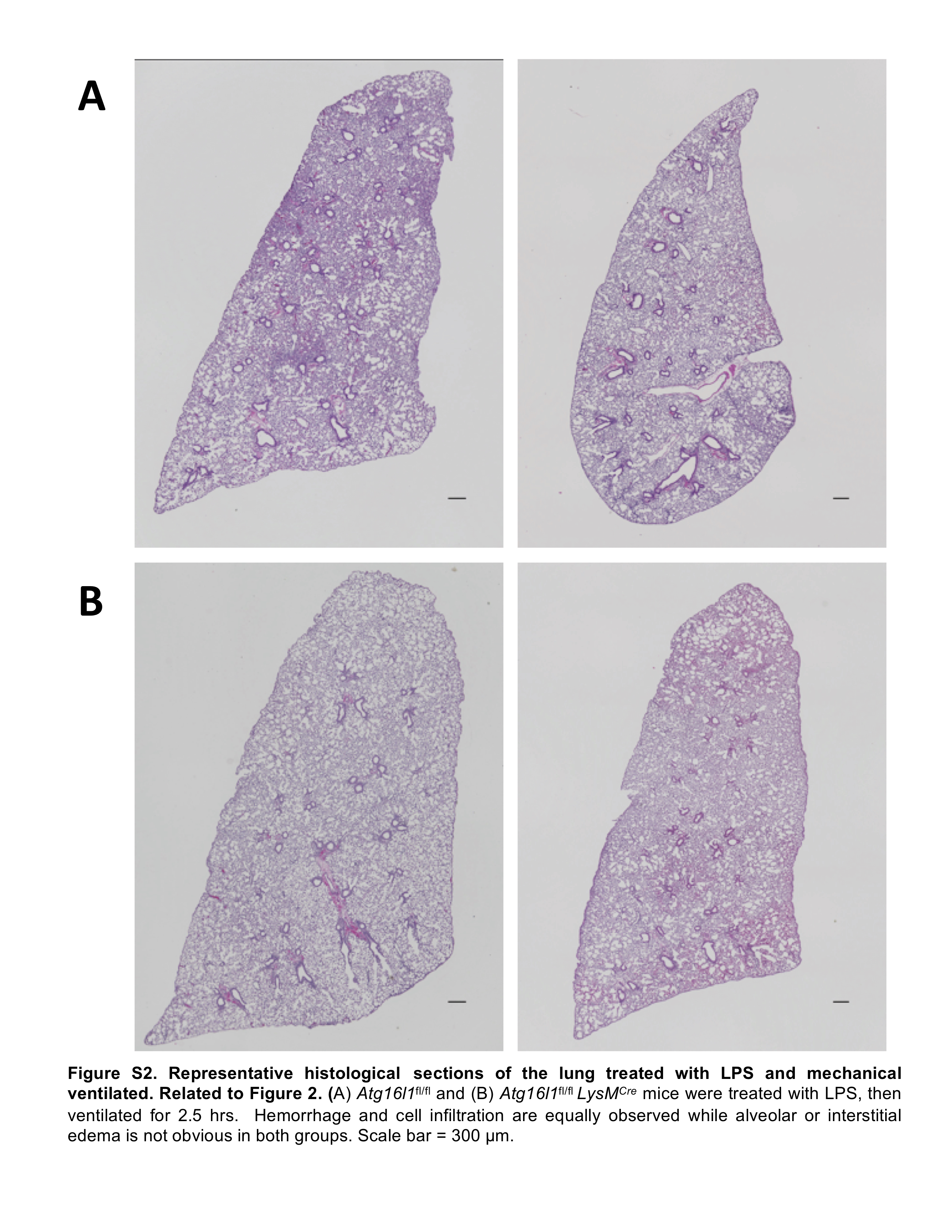

Supplement: Supplementary file 2 [file Image_2.jpg]

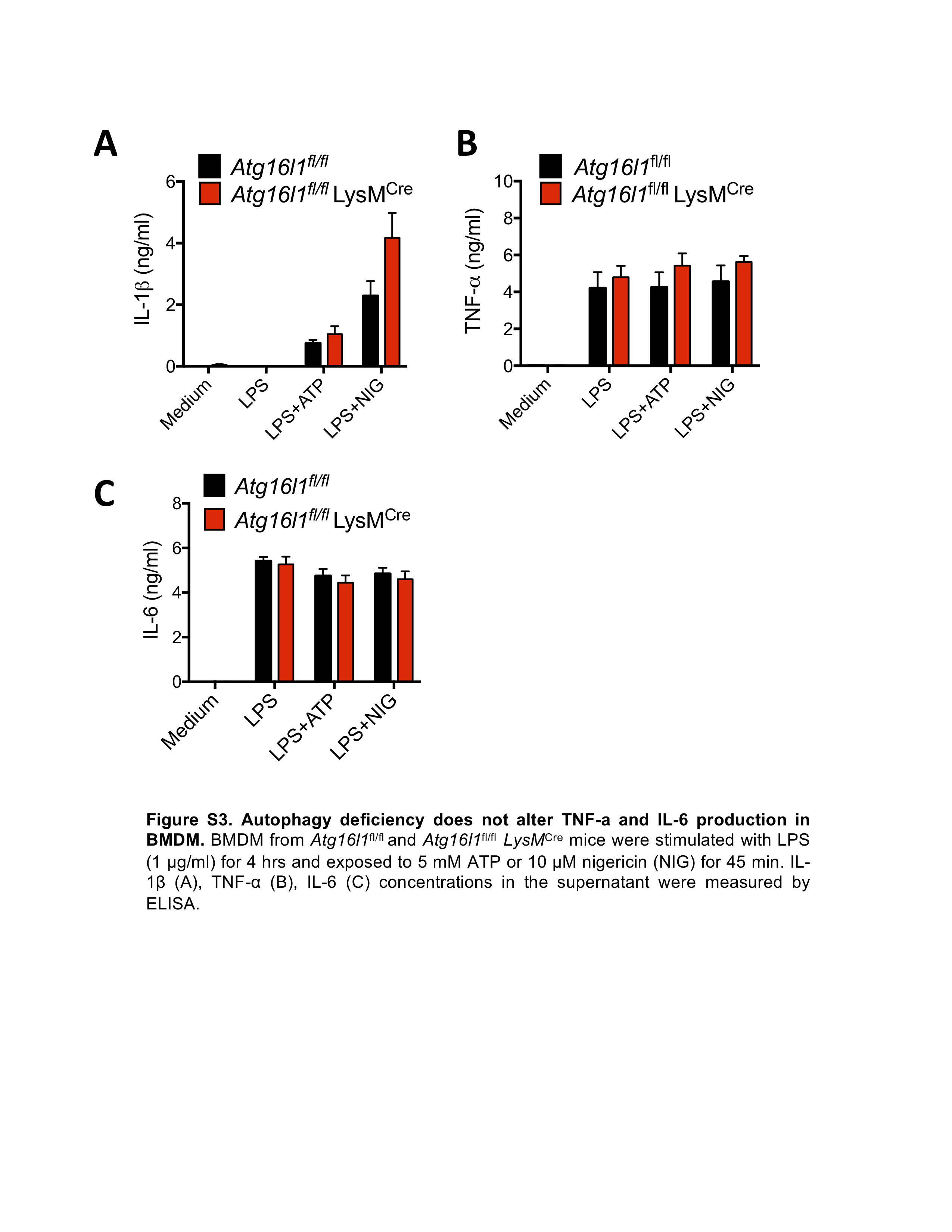

Supplement: Supplementary file 3 [file Image_3.jpg]

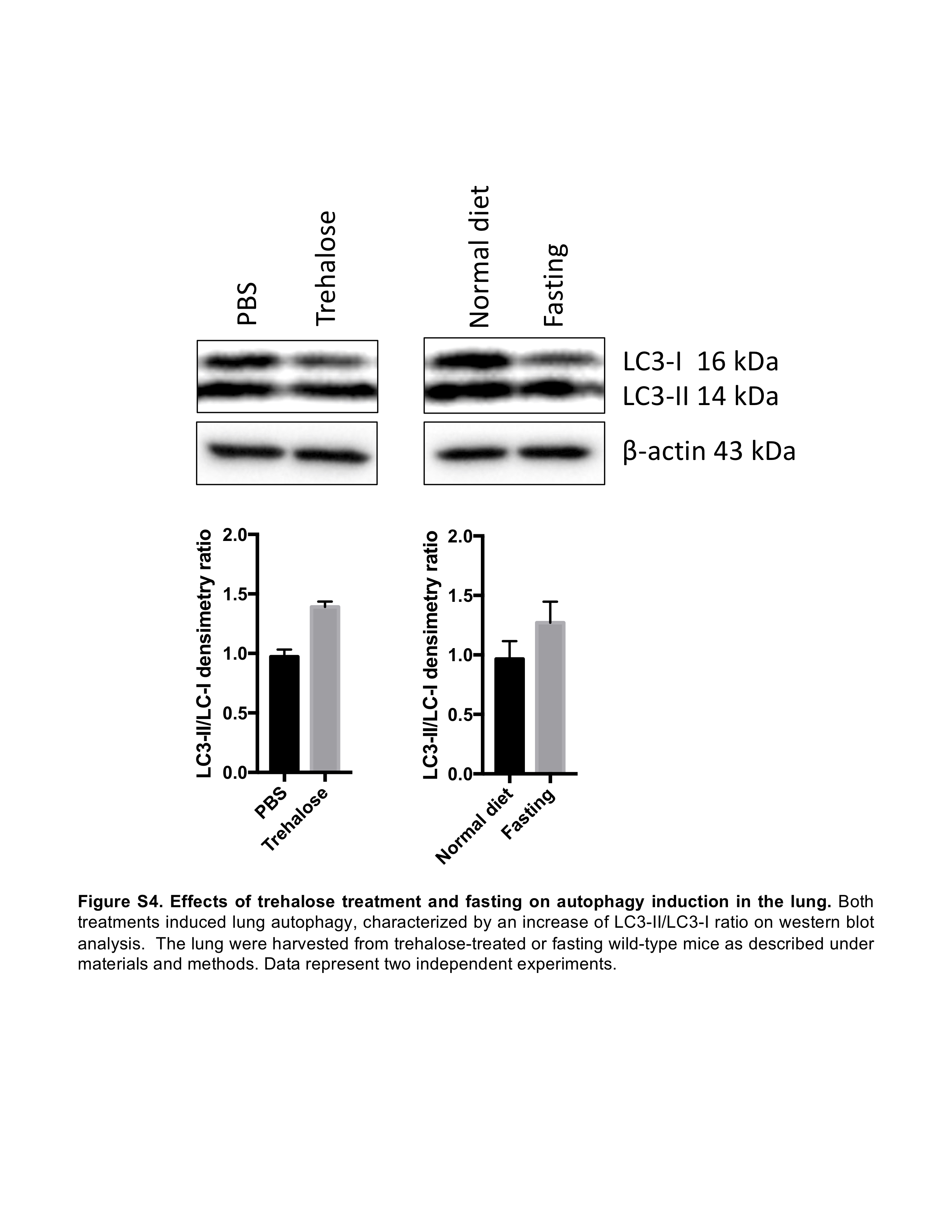

Supplement: Supplementary file 4 [file Image_4.jpg]

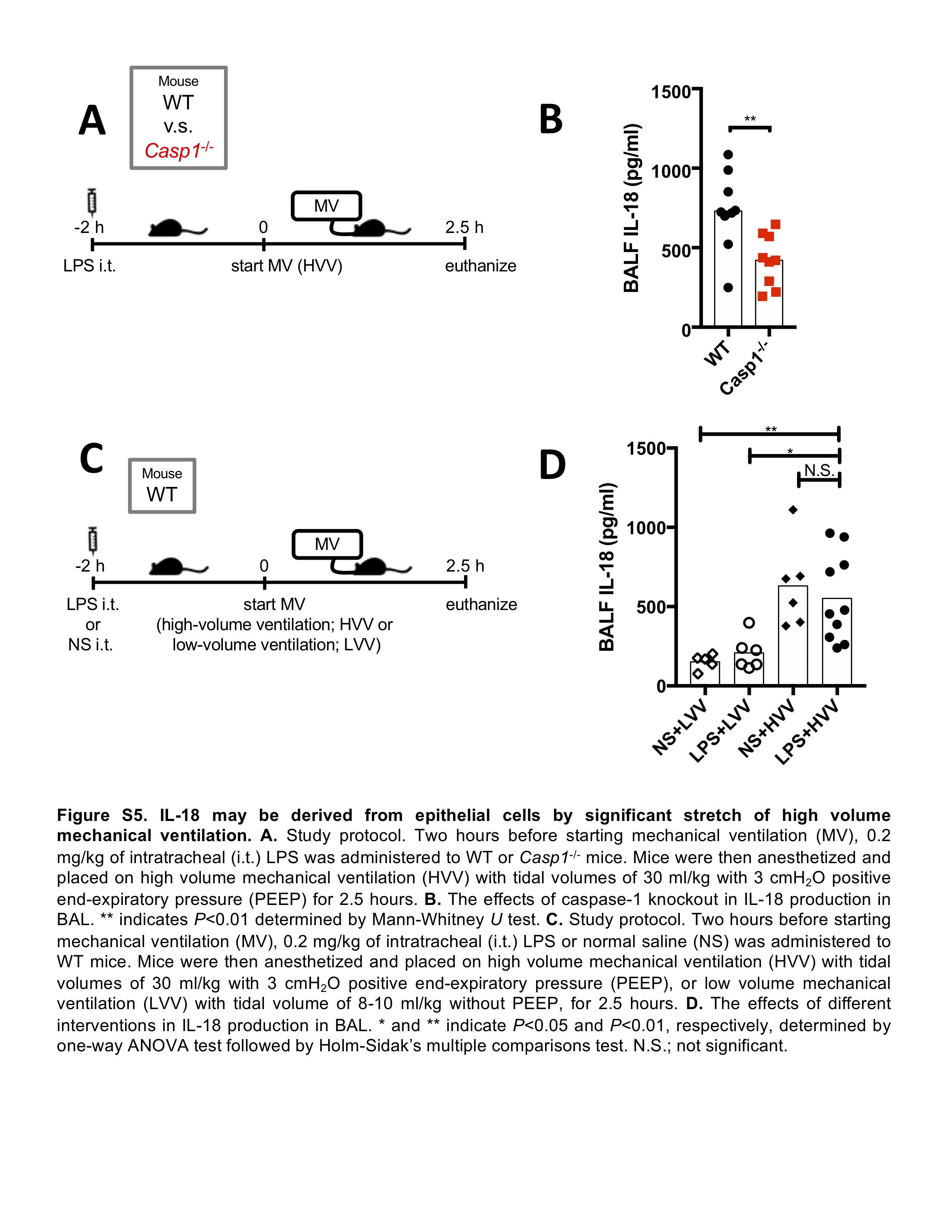

Supplement: Supplementary file 5 [file Image_5.jpg]
